# High-efficiency GaAs and GaInP solar cells grown by all solid-state molecular-beam-epitaxy

**DOI:** 10.1186/1556-276X-6-576

**Published:** 2011-10-31

**Authors:** Shulong Lu, Lian Ji, Wei He, Pan Dai, Hui Yang, Masayuki Arimochi, Hiroshi Yoshida, Shiro Uchida, Masao Ikeda

**Affiliations:** 1 Suzhou Institute of Nano-Tech and Nano-Bionics, Chinese Academy of Sciences, Suzhou Industrial Park, Ruoshui Road 398, Suzhou, China; 2 Advanced Material Laboratories, Sony Corporation, Atsugi Tec. 4-14-1 Asahi-cho, Atsugi-shi, Kanagawa, 243-0014 Japan

## Abstract

We report the initial results of GaAs and GaInP solar cells grown by all solid-state molecular-beam-epitaxy (MBE) technique. For GaAs single-junction solar cell, with the application of AlInP as the window layer and GaInP as the back surface field layer, the photovoltaic conversion efficiency of 26% at one sun concentration and air mass 1.5 global (AM1.5G) is realized. The efficiency of 16.4% is also reached for GaInP solar cell. Our results demonstrate that the MBE-grown phosphide-contained III-V compound semiconductor solar cell can be quite comparable to the metal-organic-chemical-vapor-deposition-grown high-efficiency solar cell.

## Introduction

The efficiency of III-V compound semiconductor of GaAs and GaInP solar cells continues to improve with the optimized material quality and device processing. Taking into account of the material quality only, to date, the highest performance has been realized for the materials grown by metal-organic chemical vapor deposition (MOCVD) [1-3]. It is normally believed that the efficiency of solar cell fabricated by the materials grown by molecular-beam-epitaxy (MBE) is lower than that of MOCVD growth [4,5], though MBE has been proved to be an effective tool for the basic research with its own unique advantage [6]. One of the main obstacles for the MBE-grown solar cell is considered to be the more defect states and deep centers due to the low growth temperature. In addition, the operation of phosphide-related material growth by using MBE is still thought to be a complicated task owing to its high evaporation pressure of phosphide, especially the GaInP film has been proved to be a much more suitable material for the high energy-absorption of solar spectrum than AlGaAs. Therefore, the fabrication of phosphide-related solar cell grown by MBE is scarce.

In this letter, we report the initial results of GaAs and GaInP solar cells grown by solid-state MBE. Our results demonstrate that the MBE-grown phosphide-contained III-V compound semiconductor solar cell can be quite comparable to the case of MOCVD grown.

## Experimental and results

The epitaxial growth of the solar cell material was performed by Veeco GEN20A dual chamber (Veeco Instruments, Inc., Plainview, NY, USA) all solid-state MBE equipped with a valved phosphorous cracker cell and a valved arsenic cracker cell. The typical growth rate of GaAs and GaInP is 1 μm/h, which is evidenced by XRD measurements and RHEED oscillation. The typical growth temperature of GaAs was 580°C with silicon and berylium as the n- and p-type doping source. The V/III ratio is about 30. For the GaInP growth, since the indium desorption at a high temperature will affect the indium composition, so a moderate temperature of 470°C is used in the growth. During the GaInP growth, the RHEED image shows a 2 × 1 surface reconstruction. After growth, the structures were then processed following the standard III-V solar cell device art. The cell size is 5.0 × 5.0 mm. The metal in the front grid is based on the AuGe/Ni/Au system. In order to study the effect of different shadows on the device performance, we designed two different masks with 2.1% and 8.2% shadowing areas. An antireflecting coating (ARC) layer of Si_3_N_4_/SiO_2 _was deposited on the devices. The photovoltaic current-voltage (*I*-*V*) characteristics were measured under air mass 1.5-global (AM1.5G) illumination.

**Figure 1:**
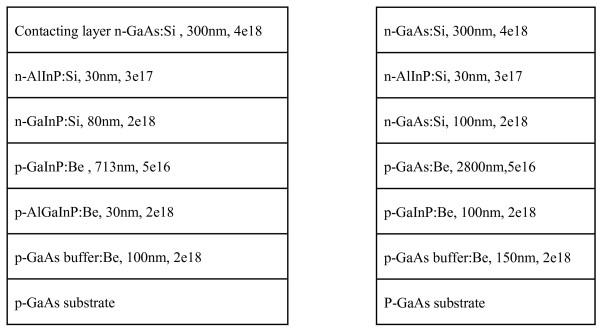
**Designed GaAs (right) and GaInP (left) single-junction solar cell structures**.

**Figure 2:**
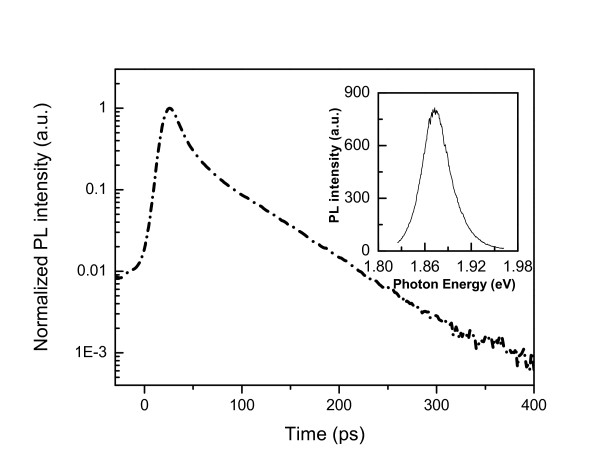
**Time evolution of normalized PL peak intensity of GaInP lattice matched grown on GaAs substrate**. With the photoluminescence peak of 1.875 eV (shown in the inset) at room temperature.

Figure 1 shows the designed GaAs and GaInP single-junction solar cell structures, respectively. For GaAs solar cell, a GaInP layer is used as the back surface field (BSF) and an AlInP layer is used as the window layer to decrease the surface recombination. The BSF and window layers are very significant since a fast surface recombination rate is observed for the III-V semiconductor in contrast to the silicon. The lattice mismatch between these layers and GaAs is less than 5 × 10^-4^. Figure 2 shows the typical time evolution of GaInP film with the photoluminescence peak of 1.875 eV (shown in the inset) at room temperature. A single exponential decay curve is observed. In addition, the decay time is independent on the detecting energy. These behaviors indicate that the emission most originates from the disordered GaInP film, since a detecting energy dependent time evolution has been observed in ordered GaInP:Si film as described in our previous study of GaInP [7].

**Figure 3:**
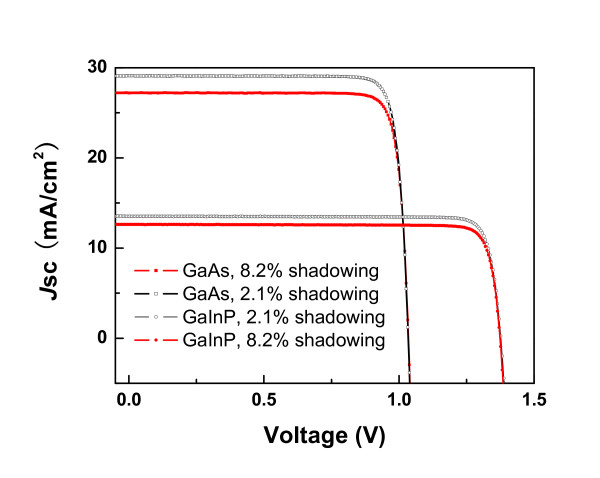
**The current-voltage (*I*-*V*) characteristics of GaAs and GaInP solar cells for different shadowing areas**.

**Figure 4:**
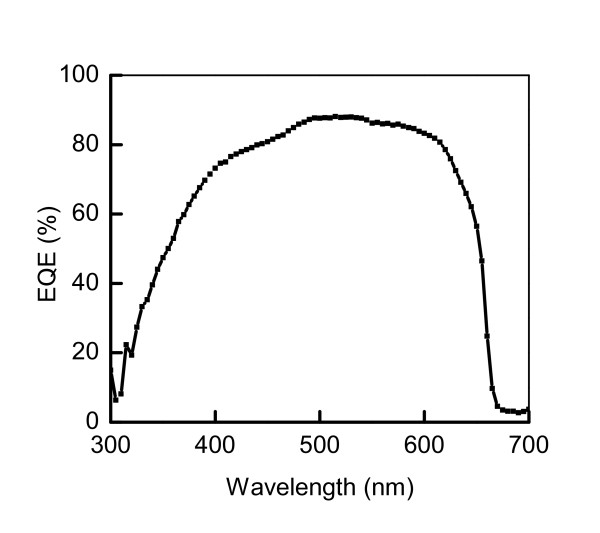
**External quantum efficiency (EQE) of the GaInP solar cell**.

The current-voltage (*I*-*V*) characteristics of GaAs and GaInP solar cells for different shadowing areas are depicted in Figure 3 under the standard solar simulator of 1.5 G. It is obviously that the larger shadowing area results in the more loss of photocurrent. However, the fill factor (FF) of the 8.2% shadowing area is a little bit larger than the case of 2.1% due to the smaller metal resistance. The calibration at 1 sun gave that the GaAs solar cell has the photovoltaic conversion efficiency of 26% with the open voltage (Voc) of 1.04 V, a short-circuit current density (*J*sc) value of 29.1 mA/cm^2^, and an FF of 86%. This efficiency is comparable with the reported value at the end of 2010 [8]. The GaInP solar cell has the best photovoltaic conversion efficiency of 16.4% with the Voc of 1.37 V, a *J*sc value of 13.5 mA/cm^2^, and a FF of 88%. The result of external quantum efficiency (EQE) of the GaInP solar cell was plotted in Figure 4. The *J*sc calculated by means of the convolution of the EQE value obtained for the device with the standard solar spectrum of AM1.5G, shows the value of 13.8 mA/cm^2^, which is almost the same as the measured value from the *I-V *characteristics.

## Discussion

Since the operation of the solar cell device includes the process of photon absorption, carriers separation, transport, and collection; therefore, many parameters affect the performance improvement of the device. However, if only taking into account of the material quality, we need to increase the diffusion length of minority carriers and decrease the recombination, such as surface recombination, radiative recombination, defect-state-related recombination, etc. In order to optimize the above factors, the most important factor is to lower the intrinsic (background) carrier density and to increase the mobility. Though the high-temperature growth by MOCVD is believed to be good to the optic-electric solar cell device due to the decrease of the defect densities and deep centers, however, the ultra-high vacuum system of MBE is good at the decreasing the background carrier density. The performance of solar cell device benefits from the high-purity material growth.

## Conclusions

By using the all solid-state MBE technique, we have fabricated the GaAs and GaInP solar cells. For GaAs single solar cell, the photovoltaic conversion efficiency of 26% at 1-sun concentration and AM1.5G is realized. Our results demonstrate that the ultra-high vacuum system of MBE is good at the decreasing the background carrier density, which is very significant to the performance of solar cells. In this case, the MBE-grown phosphide-contained III-V compound semiconductor solar cell can be quite comparable to the MOCVD-grown high-efficiency solar cell.

## Competing interests

The authors declare that they have no competing interests.

## Authors' contributions

SLL, LJ, WH, PD, and MA carried out the material growth. HY and SU performed the device fabrication. HY and MI designed the device structure and performed the statistical analysis. All authors read and approved the final manuscript.
